# A Review of the Economic Evidence of Typhoid Fever and Typhoid Vaccines

**DOI:** 10.1093/cid/ciy1122

**Published:** 2019-03-07

**Authors:** K Luthra, E Watts, F Debellut, C Pecenka, N Bar-Zeev, D Constenla

**Affiliations:** 1 International Vaccine Access Center, Department of International Health, Johns Hopkins Bloomberg School of Public Health, Baltimore, Maryland; 2 Center for Vaccine Innovation and Access, PATH, Geneva, Switzerland; 3 Center for Vaccine Innovation and Access, PATH, Seattle, Washington

**Keywords:** typhoid fever, vaccination, cost of illness, cost of vaccine delivery, cost-effectiveness analysis

## Abstract

Typhoid places a substantial economic burden on low- and middle-income countries. We performed a literature review and critical overview of typhoid-related economic issues to inform vaccine introduction. We searched 4 literature databases, covering 2000–2017, to identify typhoid-related cost-of-illness (COI) studies, cost-of-delivery studies, cost-effectiveness analyses (CEAs), and demand forecast studies. Manual bibliographic searches of reviews revealed studies in the gray literature. Planned studies were identified in conference proceedings and through partner organization outreach. We identified 29 published, unpublished, and planned studies. Published COI studies revealed a substantial burden in Asia, with hospitalization costs alone ranging from $159 to $636 (in 2016 US$) in India, but there was less evidence for the burden in Africa. Cost-of-delivery studies are largely unpublished, but 1 study found that $671 000 in government investments would avert $60 000 in public treatment costs. CEA evidence was limited, but generally found targeted vaccination programs to be cost-effective. This review revealed insufficient economic evidence for vaccine introduction. Countries considering vaccine introduction should have access to relevant economic evidence to aid in decision-making and planning. Planned studies will fill many of the existing gaps in the literature.

Typhoid fever is a common and preventable disease in low- and middle-income countries (LMICs). Varying burden estimates from different sources exist, using different modeling methods and ranging from 12 to 293 cases per 100 000 person-years due to mortality [1, 2] in regions impacted by the disease. Caused by the bacteria *Salmonella* Typhi, typhoid fever is endemic to countries in South and Southeast Asia and, more recently, in Africa, with multidrug-resistant types. Transmission is primarily through the ingestion of contaminated food or water [3].

Preventive measures include vaccination, in addition to providing access to safe water and improving hygiene and sanitation practices. Typhoid conjugate vaccines (TCVs) have recently been licensed for use in Nepal, India, and China as a single, intramuscular dose, and are indicated for use in infants at least 6 months of age [4].

The World Health Organization (WHO) Strategic Advisory Group of Experts (SAGE) on Immunization recommended the use of TCVs in the routine immunization programs of typhoid-endemic countries in October 2017. A month later, Gavi, the Vaccine Alliance, paved the way for Gavi-eligible countries to introduce TCVs in their countries with their November 2017 announcement to provide US$85 million in support [5]. By December 2017, the WHO prequalified the first typhoid conjugate vaccine, Typbar-TCV, developed by Indian pharmaceutical company Bharat Biotech, giving way for countries to purchase this vaccine through United Nations procurement agencies [6].

While understanding of the disease burden of typhoid in LMICs is growing, the economic burden of typhoid and the economic benefits of vaccination are not well understood. There is little consensus on which guidelines to adopt to estimate the costs of typhoid fever. The recent licensure of TCVs, the WHO’s SAGE recommendation, the WHO prequalification, and funding support for the introduction of TCVs into LMICs by Gavi has catalyzed renewed interest in the economic burden of typhoid and the potential cost-effectiveness (CE) of introducing TCVs into routine immunization, along with catch-up campaigns for children up to 15 years of age in endemic settings.

In this paper, we explore the existing and planned economic studies related to typhoid fever and vaccination in typhoid-endemic countries, identify gaps and limitations in the existing literature, and summarize research methodology recommendations that may enable future studies to fill these gaps.

## METHODS

We adopted a systematic approach to identify published, unpublished, and planned studies covering a 17-year period (2000–2017). Key articles representing the evidence around the cost of illness (COI) of typhoid fever, the cost of typhoid vaccine delivery, the economic benefits of typhoid vaccines, and forecasting demand for typhoid vaccines were considered. We used 4 major electronic databases (the US National Library of Medicine and the National Institutes of Health Medical [PubMed], Excerpta Medica Database [EMBASE], Elsevier’s Scopus, and The American Economic Association’s electronic bibliography [EconLit]) to locate published studies using variations of the following 3 terms: “typhoid” AND “paratyphoid” AND “econom*”. Manual bibliographic searches from relevant review papers and the WHO website, and via Google and relevant partner organizations’ websites, revealed more articles. Additional studies were identified from abstracts and presentations in the 10th International Conference on Typhoid and Other Invasive *Salmonelloses* in Uganda (April 2017), and through outreach to partner organizations (eg, The Coalition against Typhoid, the Typhoid Vaccine Acceleration Consortium, the Severe Typhoid in Africa project, and the Surveillance for Enteric Fever in Asia Project) and leading researchers in the field. Our primary focus was typhoid-endemic countries.

We removed duplicate citations and screened separately for eligibility using the title and abstract. Papers meeting the basic inclusion criteria—original studies published in English, published or conducted from 2000 onward, and that reported economic evidence of typhoid and paratyphoid fever and all typhoid vaccines from LMICs—were included in the review.

For the selected papers, we retrieved and read full-length versions. An independent reviewer extracted the following information from the selected papers: methodological approach (eg, perspective, time horizon); key findings of the study by year, country, and vaccine analyzed; and types of data used in the analysis. Selected papers were not reviewed for quality; however, their strengths and limitations were assessed. Articles that used multiple methodologies were included in more than 1 category. References were managed in Endnote [7]. All monetary values presented in this review are adjusted to the same currency and year (2016 US$) for comparison, unless otherwise specified.

## RESULTS

### Literature Search

**Figure 1.**
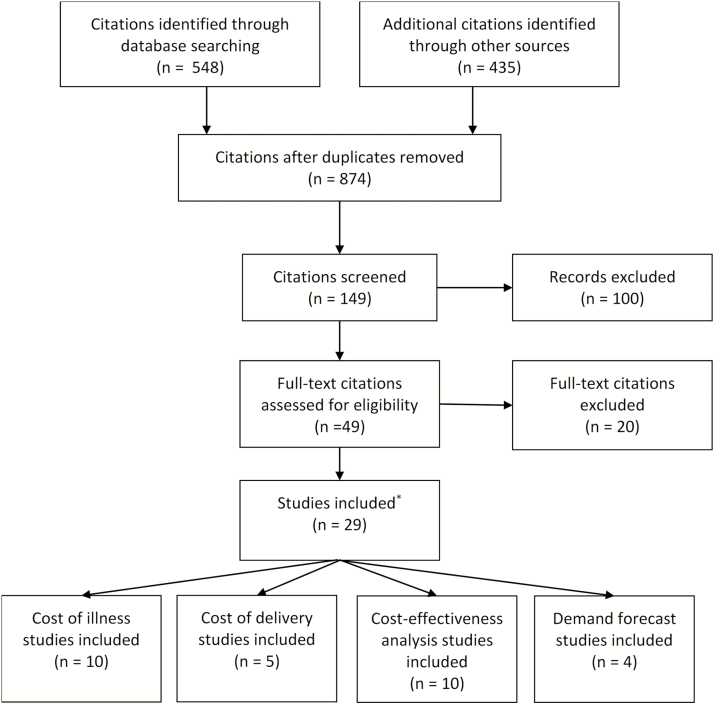
Typhoid fever economic evidence Preferred Reporting Items for Systematic Reviews and Meta-Analyses (PRISMA) flow diagram. ^a^Outreach to partner organizations resulted in 2 additional studies: 1 cost-of-illness and 1 cost-effectiveness study. This resulted in 31 studies included in the literature review.

The literature search yielded a total of 548 articles, of which only 31 met the inclusion criteria on the basis of the title and abstract. These articles were either published, unpublished, or planned studies, including 11 COI studies, 5 cost-of-delivery studies, 11 cost-effectiveness analyses (CEA), and 4 demand forecast (DF) studies (Figure 1). Nearly half of the identified evidence (n = 14) came from unpublished studies. Evidence came from 14 countries: 7 in Asia and 7 in Africa. Target cohorts ranged from all ages in COI studies to selected at-risk groups targeted for vaccination. Table 1 provides a summary of all available economic evidence.

**Table 1.** Summary of Available Economic Evidence

Type of Study	No. of Studies	Countries Studied (No. of Studies)^a^	Target Age Groups Represented (No. of Studies)^b^	Vaccines Assessed	ICER Calculated (No. of Studies)^c^
Cost-of-illness	5 studies published; 1 study unpublished; *5 studies planned*	China (1), India (3), Indonesia (1), Nepal (1), Pakistan (1), Tanzania (1), Vietnam (1), *Bangladesh (1), Burkina Faso (1), Ethiopia (1), Ghana (1), India (2), Madagascar (1), Malawi (1), Nepal (2), Pakistan (1)*	All ages (2); 0–40 years (1); >2 months (1); 2–15 years (1); 5–18 years (1); 5–60 years (1); <18 years (1); ≥18 years (1); *>9 months (1); not specified (5)*	N/A	N/A
Cost-of-delivery	1 study published; 1 study unpublished; *3 studies planned*	Not specified (1), *Malawi (1), Nepal (2)*	<18 years (2); ≥18 years (1); *not specified (3)*	ViPS, *TCV*	N/A
Cost-effectiveness and cost-benefit analysis	6 studies published; 1 study unpublished; *4 studies planned*	India (4), Indonesia (1), Kenya (1), Pakistan (1), Vietnam (2), LMICs (1), *India (1), Malawi (1), Nepal (1), LMICs (1)*	9 months (3); 9 months–5 years (3); 9 months–15 years (2); 9 months–25 years (2); ≥9 months (2); ≥2 years (4); 2–5 years (1); 2–15 years (2) 5–14.9 years (4); 6–19 years (1); ≥15 years (1); *not specified (4)*	ViPS, *TCV*	7 studies measured cost per DALY averted as an outcome; 2 studies measured cost per case averted as an outcome
Demand forecast	1 study published; 2 studies unpublished *1 study planned*	LMICs (3)*; LMICs (1)*	9 months (3); 15 months (1); 18 months (1); 1–15 years (3); 2–15 years (1); 5–15 years (1)	ViPS, Ty21a, *TCV*	N/A
Total	13 studies published; 5 studies unpublished; *13 studies planned*	China (1), Bangladesh (1), Burkina Faso (1), Ethiopia (1), Ghana (1), India (12), Indonesia (3), Kenya (1), Madagascar (1), Malawi (3), Nepal (4), Pakistan (2), Tanzania (1), Vietnam (2), LMICs (4)	All ages (2); 0–40 years (1); >2 months (1); 9 months (6); 9 months–5 years (3); 9 months–15 years (2) 9 months–25 years (2); ≥9 months (2); 15 months (1); 18 months (1); 1–15 years (3); ≥2 years (4); 2–5 years (1); 2–15 years (4); 5–18 years (1); 5–14.9 years (5); 5–60 years (1); 6–19 years (1); <18 years (3); ≥15 years (1); ≥18 years (2)	ViPS, Ty21a, TCV	7 studies measured cost per DALY averted as an outcome; 2 studies measured cost per case averted as an outcome

The text in italics indicates studies planned, to differentiate where evidence exists and where it is forthcoming.

Abbreviations: DALY, disability-adjusted life-years; ICER, incremental cost-effectiveness ratio; LMICs, low- and middle-income countries; N/A, not applicable; TCV, typhoid conjugate vaccine; Ty21a, live, attenuated oral typhoid vaccine; ViPS, Vi polysaccharide typhoid vaccine.

^a^Studies included data from multiple countries, as well as both studies focusing on selected LMICs (eg, Gavi eligible) and all LMICs.

^b^The age groups were counted separately if the study included multiple target age groups.

^c^Studies can include both ICER categories.

### Cost-of-illness Studies

COI studies measure the burden of a disease to society in monetary terms. We identified 11 COI studies, which included 5 published studies (Table 2), 1 unpublished study (Table 3), and 5 planned studies (Table 4). Studies adopted either a societal perspective or a government or public system perspective, and most included both inpatient and outpatient costs. Data collection included both retrospective and prospective methods. Existing evidence was primarily from high-incidence Asian countries, while more geographic diversity was found in planned studies. Most studies required blood culture–positive typhoid fever for inclusion in the study, although blood culture–positive paratyphoid and clinical typhoid were also included in 1 study [8]. Due to the nonspecific nature of clinical typhoid, cost estimates of blood culture–negative typhoid fever are difficult to interpret, but these figures were presented separately.

**Table 2.** Summary of Cost-of-illness Studies Published

Reference	Countries	Disease Definition	Study Participants	Study Perspective	Costs Included	Cost Sources	Results (Expressed in 2016 US$)
[8]	India	Blood culture– positive typhoid or paratyphoid; blood culture–negative with clinical typhoid	0–40 years	Societal	Direct medical, nonmedical, and indirect costs	Prospective participant interviews	- Mean total cost per episode: $126; - Mean total cost per case hospitalized: $636; - Mean total cost per outpatient case: $74; - Private and public costs are similar ($61 and $65); - Costs are highest for children (2–5 years)
[9]	Nepal	Blood culture– positive typhoid fever	All ages	Societal	Direct medical, nonmedical, and indirect costs	Qualitative interviews	- Mean direct costs per household: $92; - Mean indirect costs per household: $32; - Mean loss of income: $37; - Money borrowed: $23–284; - Total direct cost per hospitalized case: $166; - Total indirect cost per hospitalized case: $67; - Total direct cost per nonhospitalized case: $39; - Total indirect cost per nonhospitalized case: $5
[10]	China, Indonesia, India, Pakistan, Vietnam	Blood culture– positive typhoid fever	Vietnam: 5–18; China: 5–60; India: all ages; Pakistan: 2–15.	Governmental; household	Direct medical, nonmedical, and indirect costs	Patient surveys and hospital records	- Cost per case hospitalized: $159 (India), $531 (Indonesia); - Cost per nonhospitalized case: $16 (India); $82 (China); - Hospitalized case cost: up to 15% of annual household income in some settings
[11]	Tanzania	Blood culture– positive typhoid fever	>2 months	Societal	Direct costs and indirect costs	Patient records and interviews	- Mean cost per episode: $171; - Mean cost for treatment: $24; - Mean lost wages: $142
[12]	India	Widal-positive and/ or blood culture– positive typhoid fever	<18 years or ≥18 years.	Provider	Direct medical costs	Hospital records	- Average cost of treatment: children $23, adults $58, and all $26; - Hospitalized child patients incurred higher costs than hospitalized adult patients

**Table 3.** Summary of Demand Forecast Studies Published

Reference	Countries	Vaccines	Study Participants	Costs Included	Cost Sources	Results (Expressed in 2016 US$)
[13]	Typhoid endemic low and LMIC	TCV	- High-risk population and general population; - RI (9 mo.) + catch up 1–15 years; - RI (9 & 15 mo.) + catch up 1–15 years	N/A	N/A	TCV demand ranges from 40–160 million doses/year

Abbreviations: LMIC, low- and middle-income countries; N/A, not applicable; RI, routine immunization; TCV, typhoid conjugate vaccine.

**Table 4.** Summary of Upcoming Studies by Study Type

Group of Implementers	Countries	Vaccine	Disease Definition	Proposed Methods
*Cost-of-illness studies*				
V. Mogasale, D. Song, & S. Pallas	India	N/A	Not specified	Hospital-based surveillance 2017–2018 at: (1) Mumbai (urban slum served by the Grant Medical College, a tertiary-care government hospital), and (2) Pune (the King Edward Memorial [KEM] Hospital rural surveillance site, Vadu)
V. Mogasale & E. Ramani	Burkina Faso, Ethiopia, Ghana, Madagascar	N/A	Blood culture–positive typhoid/para- typhoid, iNTS, and culture-negative with clinical diagnosis	- Analytical horizon from illness onset through 12 months post-enrollment; - Facility cost estimation: cost to charge ratio; - Patient cost estimation: in-person interviews throughout duration of illness (at 1 week, 2 weeks, 1 month, 3 months, 6 months, 9 months, 12 months) using data cards to track costs between interviews
S. Pallas, N. M. Gonzalez, & T. Abimbola	Bangladesh, India, Nepal, Pakistan	N/A	Blood culture–positive typhoid fever, paratyphoid fever, and iNTS	- Analytical horizon from illness onset through 12 months post-enrollment; - Facility cost estimation: ingredients-based micro-costing; - Patient cost estimation: phone interviews when blood culture confirmed or following patient discharge (6 weeks and 12 months for cases with complications at 6 weeks), in person
N. Bar-Zeev, C. Pecenka, & F. Debellut	Malawi	N/A	Blood culture–positive typhoid fever, paratyphoid fever, and iNTS	- Analytical horizon from illness onset through 12 months post-enrollment; - Facility cost estimation: ingredients-based micro-costing; - Patient cost estimation: in-person interviews (at 1 week, 2 weeks, 1 month, 3 months, 6 months, 9 months, 12 months) using data cards to track out-of-pocket expenditures between interviews
D. Constenla, C. Garcia, & E. Watts	Nepal	N/A	Blood culture–positive typhoid fever, paratyphoid fever, and iNTS	- Analytical horizon: from illness onset through 12 months post-enrollment; - Facility cost estimation: ingredients-based micro-costing; - Patient cost estimation: in-person interviews throughout duration of illness (at days 7, 14, 30, and 90) using caregiver surveys and daily expenditure booklets to track out-of-pocket expenditures between interviews
*Cost-of-delivery studies*				
V. Mogasale, D. Song, & S. Pallas	India	TCV	N/A	Hospital-based surveillance 2017–2018 at: (1) Mumbai (urban slum served by the Grant Medical College, a tertiary-care government hospital), and (2) Pune (the KEM hospital rural surveillance site, Vadu)
C. Pecenka & F. Debellut	Malawi	TCV	N/A	- Mix of primary data collection during TyVAC impact studies associated with discussions with country Expanded Program on Immunization (EPI) teams; - Micro-costing approach: costs will be presented per activity, separated between capital and recurrent costs
D. Constenla, C. Garcia, & E. Watts	Nepal	TCV	N/A	- Mix of primary data collection during TyVAC impact studies associated with discussions with country EPI teams; - Micro-costing approach: costs will be presented per activity, separated between capital and recurrent costs
*Cost-effectiveness studies*				
J. Bilcke, M. Antillon, Z. Pieters, E. Kuylen, L. Abboud, K., M. Neuzil, A. J. Pollard, A. D. Paltiel, & V. E. Pitzer	Gavi-eligible countries	TCV	N/A	Model-based approach
V. Mogasale, D. Song, & S. Pallas	India	TCV	N/A	Hospital-based surveillance 2017–2018 at: (1) Mumbai (urban slum served by the Grant Medical College, a tertiary-care government hospital), and (2) Pune (the KEM hospital rural surveillance site, Vadu)
V. Pitzer, C. Pecenka, N. Bar-Zeev, & F. Debellut	Malawi	TCV	N/A	Model-based approach
V. Pitzer, D. Constenla, C. Garcia, & E. Watts	Nepal	TCV	N/A	Model-based approach
*Demand forecast studies*				
F. Debellut, N. Hendrix, V. Pitzer, D. Constenla, & C. Pecenka	All low- and middle-income countries	TCV	N/A	- Excel-based flexible-demand forecasting model, allowing for different delivery strategies (routine and campaigns) targeting age ranges; - User-friendly interface, allowing for easy changes of input values around delivery, dosage, coverage proxy, and introduction dates; - Scenario-building through assumptions and engagement within TyVAC and externally

Abbreviations: iNTS, invasive nontyphoid salmonellosis; N/A, not applicable; TCV, typhoid conjugate vaccine; TyVAC, The Typhoid Vaccine Acceleration Consortium.

Among the studies reviewed, there was considerable intra-country variation reported in the literature, and costs varied depending on the study perspective. Hospitalization costs were the most common cost assessed across all studies, and inpatient costs were considerably higher than outpatient costs, regardless of the study perspective. Costs per outpatient case ranged from $16 to $74 in India [8, 10] and were $39 in Nepal [9]. Inpatient costs ranged from $159 to $636 in India [8, 10]. A study in Nepal with a smaller sample size found the average cost per hospitalized case to be $233 [9]. Limited numerical data were available for African countries, but 1 study in Tanzania found the average cost per case (both inpatient and outpatient) to be $171 [11].

There were 4 studies that took a societal approach to estimating the cost of illness, each using the human capital method to calculate indirect costs. Of these, 3 found that indirect costs accounted for the majority of the total cost of illness. Variations in estimates primarily stem from the value assigned to absenteeism from work and to whether caregiver or child productivity loss were included. In 1 study that modeled the global cost of typhoid fever, a percentage of gross domestic product (GDP) per capita was used to value the time lost by caregivers and patients. The study estimated that the cost of typhoid fever in LMICs exceeded $1.3 billion, with 89% of costs comprised of indirect costs (V. Mogasale, B. Maskery, R. L. Ochiai, J. S. Lee, & T. F. Wierzba, manuscript in preparation, unreferenced, see Acknowledgments). A study in Tanzania estimated the total cost per episode at $172. When using the average wage to value the lost productivity of patients and caregivers, indirect costs accounted for nearly 80% of these costs [11]. Indirect costs were not as substantial in Nepal at the household level, where they represented only a quarter of household costs in hospitalized cases and less than 15% of total household costs in outpatient cases [9].

The evidence was mixed as to whether child or adult cases resulted in higher costs, with both outcomes observed across different settings [8, 12]. From 1995–1997, 1 study [8] collected data in an urban slum in Delhi. The authors in this study included public and private costs (direct medical costs, direct nonmedical costs, and indirect costs). Productivity losses for children were valued at one-quarter the average daily wage for 5–12 years and one-half the average daily wage for 12–19 years, which could contribute to the higher than expected COI among young adults. In contrast, in the study by Sur and colleagues [12], hospitalized pediatric patients incurred higher costs than hospitalized adult patients. However, 93% of the patients in the sample were children. This study was conducted from the public health-care provider perspective and included 2 hospitals in Kolkata, where 32.5% of population lives in slums with poor sanitation.

Some studies primarily considered treatment costs in the public sector, and private sector treatment rates and costs were under-represented [10]. Only 2 studies [11, 12] exclusively looked at public health facilities. An additional 2 studies [9, 10] surveyed both public and private health facilities, and 2 more [8, 13] used modeled costs to estimate the public costs.

### Immunization Delivery Cost Studies

Immunization delivery costs are defined as all costs required to deliver vaccines to the target population, excluding the costs of vaccines and injection supplies. These include health worker time and transport expenses to administer vaccines, among other costs. Of the 5 immunization delivery cost studies identified, only 1 was published (Table 5). The study by Lauria et al [14] adopted a governmental (public health system) perspective that included direct medical costs and the program costs assumed by the public sector to estimate the delivery costs for a Vi polysaccharide typhoid vaccine (ViPS). This study modelled the cost of delivery in a hypothetical population of 1 million (300 000 children, 700 000 adults), and evaluated 3 mass vaccination strategies (charging adults and children different [optimal] prices, charging uniform prices, and providing free vaccinations). In all of these scenarios, the median cost per vaccination was $1.74. The study found that $671 000 in government investment would avert $60 000 in public treatment costs [14].

**Table 5.** Summary of Cost-of-delivery Studies Published

Reference	Countries	Vaccines	Disease Definition	Study Participants	Study Perspective	Costs Included	Cost Sources	Results (Expressed in 2016 US$)
[14]	LMIC population	ViPS	Blood culture–positive typhoid fever (adjusted incidence rate)	Children, adults (ages unspecified)	Government and public health	Direct medical costs and direct program costs	Published literature	- Median public cost per case: ~$35 ($0–116); - In a population of 1 million (300 000 children, 700 000 adults) in an LMIC setting; - Annual public expenditure for treating typhoid is $61 000; - Cost of public vaccination program per 1 million people: $670 000

Abbreviations: LMIC, low- and middle-income countries; ViPS, Vi polysaccharide typhoid vaccine.

The cost of delivery is largely driven by the vaccination strategy utilized, which varies by the target population and vaccination setting. Apart from delivery costs, a leading driver of vaccine program costs (and hence of CE) is vaccine price. Unpublished data from a school-based vaccination campaign in Nepal, which included a donated supply of ViPS, found the greatest cost components of vaccine delivery to be advocacy and social mobilization. Beyond the cost of delivery, the issues of affordability and sustainability of typhoid vaccines are not raised in the literature. This evidence will be especially important for countries transitioning away from Gavi support.

### Cost-effectiveness and Cost-benefit Analyses

A CEA is an example of a comparative analysis that looks at the technical efficiency between 2 or more alternatives and that includes the effects and the costs of those alternatives. A CEA is distinct from a cost-benefit analysis, which assigns a monetary value to the measure of effect. We identified 11 CEAs, including 5 published studies (Table 6). The government or public health system perspective was the most common perspective adopted among the published literature, although 2 studies utilized a societal perspective [15, 16]. The majority of existing evidence is from India and South Asia. The most common outcome measure (incremental cost-effectiveness ratio) evaluated was cost per disability-adjusted life-years (DALYs) averted and cost per case averted.

**Table 6.** Summary of Cost-effectiveness and Cost-benefit Studies Published

Reference	Countries	Vaccines	Disease Definition	Study Participants	Study Perspective	Costs Included	Cost Sources	ICER	Results (Expressed in 2016 US$)
[17]	India, Kenya, Vietnam	TCV	Blood culture– positive typhoid fever (adjusted incidence rate)	5 strategies: (1) RI at 9 mos and RI at 9 mos + catch-up; (2) 9 mo–5 yrs; (3) 9 mo–15 yrs; (4) 9 mo–25 yrs; (5) ≥9 mos	Payer	Direct medical costs and vaccine costs	Published literature and government planning records	Cost per DALY averted	- At $1/dose, RI (1 dose) alone compared to no vaccination was predicted as cost-saving (Delhi, India; Vietnam), very CE (Kolkata, India: $854/DALY; Kenya: $1082/DALY), or CE (Kenya: $3138/DALY); - At $1/dose, RI + campaign was more CE than RI
[18]	Uganda	ViPS	Typhoid fever	≥2 yrs of age	Government	Direct medical costs	Published and list prices	Cost per DALY and case averted	- Cost/DALY averted: $491; - Cost/case averted: $346
[15]	Indonesia, India, Pakistan, Vietnam	ViPS	Blood culture– positive typhoid fever	5–14.9 years; 2–15 years; ≥2 years.	Societal; Government	Direct medical/ nonmedical and indirect costs	Published literature, survey, hospital records, and expert input	Cost per DALY avoided	- Net social costs for community vaccination program are very CE: $185/DALY averted (India) and $635/DALY averted (Indonesia); - School-aged vaccination (Vietnam): $4366/DALY averted
[16]	India	ViPS	Blood culture– positive typhoid fever (adjusted incidence rate)	5–14.9 years; 2–15 years; ≥2 years.	Societal	Program costs, direct nonmedical costs, and indirect costs	Published literature	Cost per DALY avoided	- Cost per DALY avoided: (1) school-based strategy (5–14 yrs), $170; (2), school-based strategy (< 15 yrs), $19; (3) community-based campaign (all ages), $526; - Mean private cost/case: $14; - Mean public cost/case: $5; - Total vaccine cost: $1.28/dose; - All 3 strategies are very CE
[19]	India	ViPS	Culture–positive typhoid/ paratyphoid, culture–negative w/ clinical typhoid	2–5 years; 6–19 years; ≥2 years.	Government	Direct medical costs, nonmedical costs, and indirect costs	Published literature, surveys, and unpublished literature	Cost per case averted	- At a per-unit vaccine cost of $1.53, the public cost/case avoided: $77 (mass vaccination), $63 (school-based vaccination), $21 (targeted vaccination program for pre-school children)
[20]	Global	TCV	Not specified	(1) RI (<1 years); (2) RI (<1 years) + campaign (5–14 years)	Societal	Direct program costs and vaccine costs	Not specified	Cost per DALY avoided	- RI may be high CE in moderate-incidence settings; - RI with school catch-up campaign highly CE in high-incidence settings

Abbreviations: CE, cost-effectiveness; DALY, disability-adjusted life-years; ICER, incremental cost-effectiveness ratio; RI, routine immunization; TCV, typhoid conjugate vaccine; ViPS, Vi polysaccharide typhoid vaccine.

In India, 2 studies performed cost-benefit analyses and found public vaccination programs using ViPSs to be a good value for money, from a public health system perspective, across a variety of age-segregated target cohorts [16, 19]. The earlier study (2004) found that mass vaccination or school-based vaccination would produce net benefits when the vaccine price is low. A preschool vaccination program would produce net benefits at all vaccine price points modeled (between $0.95 and $3.81) [19]. The second study found ViPSs would produce net benefits if user fees or the social value of life were included in the analysis. The same study found their results to be very CE from a societal perspective, using 1 times the GDP per capita per DALY averted as a threshold [16].

The parameters that most influenced CE include disease incidence, vaccine costs, and the economic benefits of disease risk reduction [10]. Across delivery platforms (campaign delivery in Uganda and a school-based delivery program in India, Indonesia, Pakistan, and Vietnam), these programs were very CE (using a threshold of 1 times the GDP per capita, per DALY averted) in all countries except Vietnam, which had a low typhoid incidence in the population studied [15, 18].

ViPSs are the most common vaccines evaluated, although 1 published study and several unpublished studies evaluate the new TCVs. Models using ViPSs accounted for herd protection in selected sensitivity analyses. A recent TCV model found that accounting for herd immunity impacts CE estimates [17]. However, there was no evidence from this study or any other study reviewed about the effects of TCVs on intestinal infection, transmission, or short- or long-term carriage.

The evidence for TCV CE is limited, but generally, studies have found vaccination of targeted populations to be CE. There was 1 study that found VIPSs are not CE in low-incidence settings [15], but results are subject to assumptions surrounding typhoid incidence and mortality [20]. An evaluation of TCV use in infant routine immunization settings in India, Kenya, and Vietnam found this vaccine to be CE in most settings and cost-saving in endemic settings [17]. (Cost-effectiveness and cost savings are calculated in the same way. To calculate the cost-effectiveness and cost savings of TCVs, it is necessary to know the total cost of the vaccine and its administration, as well as the total health consequences and economic costs averted through vaccination. Calculating the total costs averted requires information on direct medical, direct nonmedical, and indirect costs of care. These costs depend on the proportion of subjects seeking each of various levels of care and the costs of each level of care. The estimation of total costs also requires knowledge of the cost, efficacy, and effectiveness of the vaccine and its administration. To estimate the health consequences averted requires the estimation of the typhoid fever mortality rate. All calculations depend on the size of the target population [those potentially affected by typhoid fever].) However, the additional benefits gained by 1-time catch-up campaigns would be economically justified. TCVs that result in reduced treatment costs are referred to as cost-saving vaccines. If the net benefits of vaccination are sufficiently large compared to the change in costs, the vaccine is referred to as cost-effective. This same outcome was found in a recent study in the same 3 countries [17]. Another unpublished study found the vaccination of high-risk populations in LMICs to be CE, and vaccination in selected high-burden countries in South, Southeast, and Central Asia to be cost-saving (2017 workshop presentation by V. Mogasale & J. S. Lee, unreferenced, see Acknowledgments).

In a global study that compared delivery strategies, routine immunization alone was likely to be CE in moderate disease burden settings (50 cases per 100 000 annually), while adding a school-based catch-up campaign was likely to be very CE in moderate disease burden settings, with larger reductions in the number of cases and the disease burden, as compared to routine immunization alone [20].

### Demand Forecast Studies

In recent years, Accelerated Development and Introduction Plans have used DF studies to shape markets for the purpose of accelerating access to new vaccines in countries where they are needed most. We identified 3 completed DF studies, of which 1 is published (Table 3) and 2 are internal analyses by international organizations (Table 7). A 2008 forecast released by Gavi estimated the total market for all typhoid vaccines in Gavi-eligible countries over the period of 2011 to 2020 to range from 178 million doses to 497 million, depending on the target cohort and delivery platform utilized (2008 presentation produced by Gavi, the Vaccine Alliance, unreferenced, see Acknowledgments). An unpublished study by the Clinton Health Access Initiative estimated the total global market size from 2018 to 2030 will be at 743 million doses (2017 conference presentation by V. Vishwanarayan, unreferenced, see Acknowledgments), while a recent paper by Mogasale and colleagues projected demand for TCVs to range from 40–160 million doses per year, depending on the target population, delivery strategy, and year of introduction for a given country [13]. This paper focused on the public sector and estimated demand across LMICs. Target populations included routine immunization of infants and various target groups for catch-up campaigns. Introduction timing and the scope of catch-up campaigns are the primary drivers of demand.

**Table 7.** Summary of Unpublished Data by Study Type

Reference^a^	Countries	Vaccines	Disease Definition	Study Participants	Perspective	Costs Included	Cost Sources	ICER Utilized	Results (2016 US$)
*Cost-of-illness studies*									
V. Mogasale, B. Maskery, R. L. Ochiai, J. S. Lee, & T. F. Wierzba, manuscript in preparation, unreferenced	LMICs	N/A	Typhoid fever adjusted for low sensitivity of diagnostics	All ages	Societal	Direct medical costs and productivity losses	Published literature, open access databases, and unpublished data	N/A	- Total annual treatment costs estimated to be $141 million in direct costs and 1.2 billion in productivity loss; - High-risk areas, (eg, southern Africa, Eastern Asia, and Southeastern Asia contributed disproportionately to treatment costs); - Average cost per episode was $114; average cost per outpatient episode was $92; average cost per inpatient episode was $421
*Cost-of-delivery studies*									
2012 summary results by V. Mogasale, unreferenced	Nepal	ViPS	Not specified	School children (ages not specified)	Provider	Direct medical and program costs	Unpublished data	N/A	- Cost per dose delivered: $7.78
*Cost-effectiveness studies*									
2017 workshop presentation by V. Mogasale & J. S. Lee; unreferenced	LMICs	TCV	Blood culture–confirmed typhoid fever (adjusted incidence rate)	- 0–4 years; - 5–14 years; - ≥15 years	Government	Program costs and wastage	Published literature and unpublished data	Cost/ DALY averted	- Vaccinating high risk: very CE; for countries in SE Asia, South Asia, and Central Asia, vaccinating population cost saving; - RI + booster dose strategies are less CE than single-dose RI strategies
*Demand forecast studies*									
2008 presentation produced by Gavi, the Vaccine Alliance, unreferenced	Gavi-eligible countries	ViPS, live oral typhoid vaccine; TCV and other typhoid vaccines under development	N/A	(1) Mass campaign: 2–15 years; 5–15 years; (2) RI (<1 years) and catch-up campaign (1–15 years); (3) RI (<1 years)	N/A	Direct program costs and direct nonprogram costs	WHO, published literature, other sources (unspecified)	N/A	- Total market (2011–2020); - Campaign 2–15 years: 497 million doses; - Campaign 5–15 years: 453 million doses; - Routine vaccination: 178 million doses
V. Vishwanarayan, unpublished observations	Gavi/LMIC	TCV	N/A	- RI (9 months, 18 months); catch-up campaign (1–15 years)	Government	N/A	N/A	N/A	- Total market size 2020–2030: 734 million doses; - Peak demand in 2023 with 102 million doses

Abbreviations: DALY, disability-adjusted life-years; ICER, incremental cost-effectiveness ratio; LMIC, low- and middle-income countries; N/A, not applicable; RI, routine immunization; SE, southeast; TCV, typhoid conjugate vaccine; ViPS, Vi polysaccharide typhoid vaccine; WHO, World Health Organization.

^a^Unpublished studies are referenced in Acknowledgments.

## DISCUSSION

At the global level, our understanding of the economic burden of typhoid fever is largely informed by evidence from a selected number of endemic countries, primarily in South and Southeast Asia and, more recently, in Africa. Moreover, a significant portion of the existing evidence comes from unpublished studies, which makes it difficult to access the full economic evidence on disease burden and vaccination. Several planned studies, however, intend to evaluate the costs of typhoid fever and the economic benefits of TCVs, which will help fill this void.

The COI papers reviewed present a wide variation of cost estimates for typhoid fever, due to differences in case definitions, sample populations, data sources, discount rates, and other factors. A common limitation noted across the COI evidence was the limited sample size included in each study, potentially reducing the generalizability of results and lack of precision of the estimates, which may account for the substantial inter-study variance [9, 11, 12]. The narrow timeframe of the studies also limits the generalizability of results, as seasonality and year-to-year fluctuations of typhoid fever are generally not captured in the studies. This limitation extends to the costs associated with hospitalization rates that were observed during the study periods [12].

Several cost categories (direct medical, direct nonmedical, and indirect costs) are not included in the literature. For example, real drug prices and the prices of capital assets were not available in some of the papers [12]. Opportunity costs, such as a typhoid patient utilizing a hospital bed that could be allocated to a sicker patient, are not currently included [8]. Household costs and indirect costs also comprise a major economic component of the cost of illness, yet were largely excluded from existing studies. In studies that included household costs, the “cost of costs”—such as transaction costs or interest payments incurred by households to afford treatment costs—was not considered [10]. Other indirect costs borne by patients and households, such as behavior change requirements to address or prevent the illness, were not considered [8]. In addition, data on households’ willingness to pay for the prevention or treatment of typhoid are largely not included [8]. Some of these limitations are addressed directly in planned studies. For example, we identified several studies that are considering the inclusion of out-of-pocket payments to have a better understanding of the burden of typhoid fever at the household level.

The existing cost-of-delivery evidence was limited to 2 studies: 1 school-based pilot program in Lalitpur, Nepal (2012 summary results by V. Mogasale, unreferenced, see Acknowledgments) and a model generalized to LMIC contexts [14]. An important limitation of the evidence generated by the pilot program is that it did not account for shipping and wastage costs, as these were provided by donation, limiting the direct validity of the results to real-world settings (2012 summary results by V. Mogasale, unreferenced, see Acknowledgments). In addition, neither study included the possible impact or extent of indirect herd protection, which may influence the total number of cases and, therefore, public treatment costs avoided [14]. Upcoming studies may address some of these gaps by accounting for (1) a greater scope of programmatic and delivery costs across a variety of delivery settings and countries, and (2) TCV specifications in terms of dosing schedules, target populations, and delivery strategies.

Available evidence suggests vaccination is highly CE, or CE in moderate- to high-burden settings, but this evidence primarily evaluated older ViPSs and vaccinations in high-burden settings, which might be misleading in a broader context. More evidence is needed on the CE of new TCVs. In addition, as most studies adopted a narrow (governmental) perspective, several cost differences were not addressed in the CE literature, including transportation costs, lost productivity, and out-of-pocket expenditures, which are potentially catastrophic in certain settings [18]. When the costs of waiting for vaccinations were included, these were not informed by data [15].

The assumed burden of disease used in published CEA models may be lower than in real-world settings, as studies focused on blood culture–positive cases and assumed only hospitalized cases died [17, 19]. In addition, differences may exist in disease burdens or disease risks between those populations able to access treatment and those who lack access [15]. Such differences would not be accounted for if cost inputs are used from COI studies that exclusively recruit participants within treatment settings. These limitations likely resulted in conservative, incremental, cost-effectiveness ratio estimates. Including non–blood culture–confirmed cases in CEA models would cause typhoid vaccines to appear more cost-effective. In addition, disease transmission models were limited by intra-country and inter-country differences in disease burden, and a limited understanding of age-specific disease incidences [20]. Disease incidence is a leading determinant of CE, and increased understanding of local disease burdens can help inform future CE studies. Unlike other, more common diseases that result in death, like pneumonia or diarrhea, typhoid fever has only a moderate mortality impact. The economic considerations of TCV introduction are, thus, more relevant.

CE is typically underestimated, due to the uncertainty of the inputs. Typhoid vaccines will remain cost-effective so long as disease incidence is high. The only instance when vaccines are not cost-effective is when the incidence is found to be low. A cost-effectiveness analysis measures health gain by the number of averted deaths or events. The higher the incidence or mortality in a population, as found in high-risk populations, the more disease cases will be prevented. To set a minimum level of typhoid incidence for any given area that results in cost-effectiveness, a threshold analysis could be performed. A threshold analysis is a benchmark that can be used to set a minimum level of typhoid incidence for any given area that results in cost-effectiveness. This becomes important when incidence data is uncertain.

Another major limitation in CE is the lack of accounting for herd protection, waning vaccine efficacy, and natural immunities within communities in many disease transmission models [16, 18]. The limited timeframes adopted in several studies (eg, 3 years) prevented analyses of incorporating the need for revaccination into CE estimates in studies that modeled vaccination with ViPSs, where protection is assumed to last for only 3 years. There was also limited information about antimicrobial resistance and its associated costs. Future CE studies may address some of these gaps through longer study durations, the inclusion of vaccine program costs, and a better understanding of vaccine effectiveness and herd protection associated with the new TCVs.

DF studies focused on older typhoid vaccines (Typbar, licensed in India; 2008 presentation produced by Gavi, the Vaccine Alliance, unreferenced, see Acknowledgments), and only 1 published study estimated the demand for new TCVs [21]. Uncertainties in DF parameters are not well reported in existing models. For example, the optimal number of doses and boosters needed and targeting strategies are all subject to change and could render current demand estimates quickly outdated. In addition, models do not account for factors that influence demand at the country level, such as reduced disease transmission through economic growth, improved infrastructure, and better hygiene and sanitation practices [13]. The DF study concurrently published in this volume addresses some of these limitations, as the study accounts for updated WHO recommendations regarding the use of typhoid vaccines [21]. Moving forward, DF studies for new TCVs will need to consider new product information, WHO recommendations, Gavi eligibility and transition scenarios, and competing vaccination and other health priorities for countries. Models should be flexible and easy to adapt, to account for uncertainty and the evolving landscape around key input variables.

Key conditions need to be determined and met in order to make vaccine introduction decisions. The public health benefit of the vaccine (does the vaccine reduce disease events or deaths?) is key, as is the safety of the vaccine, both individually and on the population level. Ideally, countries should estimate their own typhoid burden and the costs of typhoid fever and typhoid vaccination, as both disease burden and costs are context-specific. However, this is not always possible. Once the public health benefit of the vaccine and the safety profile is established, an economic argument can be made about the vaccine’s cost-effectiveness. Generally, there are 3 potentially advantageous types of economic profiles for vaccines, based on the relative comparison of costs and health or economic benefits. The strongest profile would be a vaccine that results in medical cost savings that exceed the costs of the product (cost-saving). In a second scenario, the net medical costs (vaccine cost - medical cost savings) may generate an acceptable health benefit (often expressed in DALYs; cost-effectiveness). Third, in a cost-benefit analysis, the cost of a vaccine may exceed the resulting medical cost savings, but the combination of productivity gains (or other cost savings) and medical cost savings may be sufficient to cover its costs (net benefit). In order to estimate the true cost-effectiveness, and facilitate generalizations to other settings, key variables influencing the cost-effectiveness ratio need to be identified, including the immunization coverage, vaccine efficacy, target population, availability of infrastructure, and disease burden. Incorporation of these key variables into an economic model can facilitate an evaluation of the cost-effectiveness of the typhoid vaccine in different settings.

In summary, greater understanding of the disease burden and economic costs of typhoid fever can help decision-makers determine the public health priority of devoting additional resources to prevention and treatment in endemic countries. Evidence about the economic benefits of TCVs can assist policymakers in making informed introduction decisions. DF studies can assist donors and vaccine manufacturers with supply decisions and market-shaping strategies. A few suggestions for ways to improve research methodology for future economic studies are presented in Table 8.

**Table 8.** Research Considerations by Study Type

Cost-of-illness Studies		Cost-of-delivery Studies		Cost-effectiveness Studies		Demand Forecasting Studies	
Cost considerations	Disease considerations	Cost considerations	Disease considerations	Cost considerations	Disease considerations	
- Adopt a societal perspective; - Improve the generalizability of results through selection of participants and costs (eg, use national averages for wage rates); - Account for different costs related to treatment-sensitive and treatment-resistant cases; - Transaction costs should be included in costs to households; - Utilize actual costs of drugs and capital assets, rather than list prices, when available; - Include treatment costs from outpatient and inpatient care; - Incorporate household willingness-to-pay estimates	- Include laboratory-confirmed typhoid fever cases, as misdiagnoses could reduce costs or increase costs; - Increase follow-up time to capture long-term impact of typhoid fever; - Excluding non–blood culture–confirmed cases may miss a large segment of the burden of typhoid fever	- Include vaccine supply and procurement costs in all studies, even if vaccine product is donated; - Use the WHO/International Vaccine Institute (IVI) cost of delivery tool to ensure consistency of cost data reported; - Consider issues of affordability and sustainability of TCV through budget impact analysis	- Include herd protection when accounting for public program costs and cost savings	- Adopt a societal perspective to capture the full economic value of vaccination; - Include geographically representative cost data to increase generalizability; - Indirect costs to households should also include data-based travel and waiting-time costs for vaccination and treatment; - Program delivery costs should reflect the delivery platform (eg, school-based delivery vs. community-based delivery); - CE comparators can be expanded beyond no vaccination or different vaccination strategies to include other typhoid prevention and reduction measures, as well as other priority disease areas; - Account for different households’ willingness to pay thresholds in different settings	- Incorporate flexibility into models to account for different risk levels and outbreak scenarios (eg, when vaccination occurs within an outbreak context); - Include potential impact of antimicrobial resistance; - Include geographically representative epidemiological data to increase generalizability of results; - Adjust models to account for increased awareness and potential for early treatment –seeking behavior among study participants	- Model expected introduction process at country level as closely as possible, particularly in countries that typically implement phased introductions; - Models should incorporate ability to adjust parameters based on multiple scenarios and evolving information, including multiple products, multiple delivery platforms, and multiple supply scenarios; - Account for anticipated country-level transitions from Gavi funding support to self-financing, as this may shift the demand curve

Abbreviations: CE, cost-effectiveness; TCV, typhoid conjugate vaccine; WHO, World Health Organization.

## CONCLUSIONS

The findings of this study underscore the importance of typhoid fever as a global public health problem. With typhoid fever, the decision for countries hinges on CE evidence. A TCV is a regional vaccine that targets a large population, with a nonuniversal market. A TCV requires significant investment in research and development; regions that are typhoid-endemic may be less able to afford the vaccine. With other vaccines, knowing they are CE and affordable is helpful. With a TCV, the economic argument is critical. Early strategic planning is needed to support the decision-making for and implementation of TCVs in preventing typhoid infections.

## Notes

The current review paper is a sub-study of The Typhoid Vaccine Acceleration Consortium (TyVAC), led by Professor Kathleen Neuzil (ethics reference OxTREC 15–17). TyVAC is a partnership between the Center for Vaccine Development at the University of Maryland School of Medicine, the Oxford Vaccine Group at the University of Oxford, and PATH, an international nonprofit that aims to accelerate the introduction of new typhoid conjugate vaccines as part of an integrated approach to reduce the burden of typhoid in countries eligible for support from Gavi.

***Acknowledgments.*** The authors thank Ms So Yeon Kang and Dr Cristina Garcia for assistance with the initial literature search; Professor Andrew Pollard, Dr Katherine Theiss-Nyland, and Ms Rachel Colin-Jones for reviewing an early draft of the manuscript; Dr Vittal Mogasale and Dr Sarah Pallas for sharing details of their upcoming typhoid fever economic field studies (Severe Typhoid in Africa and Surveillance for Enteric Fever in Asia Project); and Dr Virginia Pitzer for sharing her perspective on typhoid modeling and information about her forthcoming cost-effectiveness analysis publications. They thank the following researchers, who provided material from and information about their unpublished works: Ms Vinita Vishwanarayan (“Creating a sustainable vaccine landscape for typhoid fever,” presented at the 10th International Conference on Typhoid and other Salmonellosis, Kampala, Uganda, 2017) and Dr Vittal Mogasale (supplementary data from a school-based cost-of-delivery study in Lalitpur, Nepal; the unpublished manuscript “An estimation of economic burden of typhoid fever in low and middle income countries”; and the workshop presentation “Cost-effectiveness analysis of typhoid conjugate vaccine by global regions using static model [preliminary results based on 2014 analysis],” presented at the Typhoid Model Comparison Workshop in New Haven, Connecticut, 2017). They thank Gavi, the Vaccine Alliance, for granting public access to the 2008 Vaccine Investment Strategy Typhoid Analysis.

***Financial support.*** This publication is based on research funded in part by a grant from the Bill & Melinda Gates Foundation (OPP1151153).

***Supplement sponsorship.*** This supplement is sponsored by the Center for Vaccine Development and Global Health (CVD) at the University of Maryland School of Medicine.

***Potential conflicts of interest.*** D. C., N. B.-Z., C. P., F. D., and E. W. receive funding from The Typhoid Vaccine Acceleration Consortium (TyVAC) study. K. L. received funding from TyVAC at the time the literature review was conducted. All authors have submitted the ICMJE Form for Disclosure of Potential Conflicts of Interest. Conflicts that the editors consider relevant to the content of the manuscript have been disclosed.

## References

[1] Antillón M, Warren JL, Crawford FW, et al The burden of typhoid fever in low- and middle-income countries: A meta-regression approach. PLOS Negl Trop Dis 2017; 11:e0005376. 28241011 10.1371/journal.pntd.0005376 PMC5344533

[2] Kim JH, Mogasale V, Im J, Ramani E, Marks F Updated estimates of typhoid fever burden in sub-Saharan Africa. Lancet Glob Health 2017; 5:e969. 28911760 10.1016/S2214-109X(17)30328-5

[3] World Health Organization. Background document: The diagnosis, treatment and prevention of typhoid fever. Geneva, Switzerland: World Health Organization, 2003.

[4] Sabin Vaccine Institute. First typhoid conjugate vaccine achieves WHO prequalification, a key step in protecting children and reducing the burden of typhoid 2018 Jan 3 Available at: http://www.coalitionagainsttyphoid.org/first-typhoid-conjugate-vaccine-achieves-who-prequalification-a-key-step-in-protecting-children-and-reducing-the-burden-of-typhoid/. Accessed January 30 2018.

[5] Gavi, the Vaccine Alliance. Millions of children set to be protected against typhoid fever [Press release] 2017 Available at: https://www.gavi.org/library/news/press-releases/2017/millions-of-children-set-to-be-protected-against-typhoid-fever/. Accessed December 1 2017.

[6] World Health Organization. Typbar TCV® from Bharat Biotech, world’s first typhoid conjugate vaccine prequalified by WHO [Press release] Available at: http://www.who.int/medicines/news/2017/Bharat-Biotech-TypbarTCV-WHO-PQ-Press-Release-Global-Final.pdf?ua=1. Accessed January 30 2018.

[7] Clarivate Analytics. Endnote. Version X8 [software] 2016 Available at: https://endnote.com/. Accessed August 15 2017.

[8] Bahl R, Sinha A, Poulos C, et al Costs of illness due to typhoid fever in an Indian urban slum community: implications for vaccination policy. J Health Popul Nutr 2004; 22:304–10. 15609783

[9] Kaljee LM, Pach A, Garrett D, Bajracharya D, Karki K, Khan I Social and economic burden associated with typhoid fever in Kathmandu and surrounding areas: a qualitative study. J Infect Dis 2017; 218(Suppl):S243–9. 10.1093/infdis/jix122 PMC6226633 28973415

[10] Poulos C, Riewpaiboon A, Stewart JF, et al; DOMI Typhoid COI Study Group Cost of illness due to typhoid fever in five Asian countries. Trop Med Int Health 2011; 16:314–23. 21223462 10.1111/j.1365-3156.2010.02711.x

[11] Riewpaiboon A, Piatti M, Ley B, et al Cost of illness due to typhoid fever in Pemba, Zanzibar, East Africa. J Health Popul Nutr 2014; 32:377–85. 25395900 PMC4221443

[12] Sur D, Chatterjee S, Riewpaiboon A, Manna B, Kanungo S, Bhattacharya SK Treatment cost for typhoid fever at two hospitals in Kolkata, India. J Health Popul Nutr 2009; 27:725–32. 20099755 10.3329/jhpn.v27i6.4323 PMC2928117

[13] Mogasale V, Ramani E, Park IY, Lee JS A forecast of typhoid conjugate vaccine introduction and demand in typhoid endemic low- and middle-income countries to support vaccine introduction policy and decisions. Hum Vaccin Immunother 2017; 13:2017–24. 28604164 10.1080/21645515.2017.1333681 PMC5612352

[14] Lauria DT, Maskery B, Poulos C, Whittington D An optimization model for reducing typhoid cases in developing countries without increasing public spending. Vaccine 2009; 27:1609–21. 19146902 10.1016/j.vaccine.2008.12.032

[15] Cook J, Jeuland M, Whittington D, et al; DOMI Typhoid Economics Study Group The cost-effectiveness of typhoid Vi vaccination programs: calculations for four urban sites in four Asian countries. Vaccine 2008; 26:6305–16. 18835415 10.1016/j.vaccine.2008.09.040

[16] Cook J, Sur D, Clemens J, Whittington D Evaluating investments in typhoid vaccines in two slums in Kolkata, India. J Health Popul Nutr 2009; 27:711–24. 20099754 10.3329/jhpn.v27i6.4319 PMC2928108

[17] Antillón M, Bilcke J, Paltiel AD, Pitzer VE Cost-effectiveness analysis of typhoid conjugate vaccines in five endemic low- and middle-income settings. Vaccine 2017; 35:3506–14. 28527687 10.1016/j.vaccine.2017.05.001 PMC5462484

[18] Carias C, Walters MS, Wefula E, et al Economic evaluation of typhoid vaccination in a prolonged typhoid outbreak setting: the case of Kasese district in Uganda. Vaccine 2015; 33:2079–85. 25712333 10.1016/j.vaccine.2015.02.027 PMC8856016

[19] Poulos C, Bahl R, Whittington D, Bhan MK, Clemens JD, Acosta CJ A cost-benefit analysis of typhoid fever immunization programmes in an Indian urban slum community. J Health Popul Nutr 2004; 22:311–21. 15609784

[20] Lo NC, Gupta R, Stanaway JD, et al Comparison of strategies and incidence thresholds for Vi conjugate vaccines against typhoid fever: a cost-effectiveness modeling study. J Infect Dis 2018; 218(Suppl 4):S232–42. 29444257 10.1093/infdis/jix598 PMC6226717

[21] Debellut F, Hendrix N, Pitzer V, et al Forecasting demand for the typhoid conjugate vaccine in low- and middle-income countries. Clin Infect Dis 2018. 10.1093/cid/ciy1076 PMC6405267 30845321

